# Rosa × damascena Herrm. essential oil: anti-tyrosinase activity and phytochemical composition

**DOI:** 10.3389/fphar.2024.1451452

**Published:** 2024-09-11

**Authors:** Qiuyan Wu, Wanting Fang, Hao Liu, Zhong Liu, Xuetao Xu

**Affiliations:** ^1^ School of Pharmacy and Food Engineering, Guangdong Provincial Key Laboratory of Large Animal Models for Biomedicine, Wuyi University, Jiangmen, China; ^2^ College of Life Science and Technology, Jinan University, Guangzhou, China

**Keywords:** Rosa × damascena Herrm., essential oil, anti-tyrosinase activity, inhibition mechanism, phytochemical composition

## Abstract

Tyrosinase is a key enzyme in melanin synthesis, and its natural inhibitors are receiving increasing attention. *Rosa × damascena* Herrm. essential oil (RDEO), as important functional metabolites, was widely known due to its biological activities. But its tyrosinase inhibitory activity has not been detailed investigated. Therefore, in this paper, RDEO was comprehensively investigated the tyrosinase inhibitory, followed by the phytochemical composition analysis. Activity screening results showed that RDEO exhibited effective anti-tyrosinase activity and was a reversible and mixed-type inhibitor. CD assay results revealed that RDEO could affect the conformation of tyrosinase to reduce the activity. In B16F10 cells, RDEO (25–100 μg/mL) could inhibit intracellular tyrosinase activity and decrease melanin content. Finally, GC-MS analysis of RDEO found that citronellol (21.22%), geraniol (14.1%), eicosane (11.03%), heneicosane (6.65%) and 1-nonadecene (5.16%) were its main phytochemical compositions. This study provided data support for *Rosa × damascena* Herrm. essential oil as one potential natural tyrosinase inhibitor and its applications in cosmetics and medicine.

## 1 Introduction

Tyrosinase is a copper-containing enzyme that is naturally occurring in various life forms, including fungi, plants, and animals (Romagnoli et al., 2022; Moreiras et al., 2020). It plays a pivotal role in melanin synthesis by facilitating the conversion reaction of L-tyrosine into L-3,4-dihydroxyphenylalanine (L-DOPA) and subsequent reaction of L-DOPA to *o*-quinone (Su et al., 2023; Nasab et al., 2023). The formation of melanin pigments is further elaborated through a series of complex enzymatic reactions, which include the cyclization of DOPA-quinone and the oxidative polymerization of the resulting indole compounds (Pillaiyar et al., 2018; Najafi et al., 2023). This melanogenesis process is not only crucial for the pigmentation of human skin and hair, but also serves as a vital protective barrier against the detrimental effects of ultraviolet radiation (Ielo et al., 2019; Roberts et al., 2015). Stimulating factors such as specific medications, ultraviolet exposure, and aging process result in pigmentary disorders like melasma, freckles, age spots, and solar lentigines (Chortani et al., 2022; Hassan et al., 2023). Moreover, irregularities in melanin distribution and concentration are sometimes indicative of underlying un-health conditions.

In the quest for effective treatments for hyperpigmentation, researchers have explored the lightening effects of various chemical agents (Li et al., 2021). Given that tyrosinase is central to melanin production, inhibiting its activity always is the key strategy in developing agents for skin lightening (Wang et al., 2023). Several compounds with the potential to inhibit melanogenesis are utilized in cosmetic and medicine industries (He et al., 2023). However, most of these agents fall short of meeting the stringent criteria of clinical efficacy, specificity, minimal toxicity, and chemical stability, and may also be associated with adverse side effects (Xue et al., 2023; Barros et al., 2023).

Therefore, there is a growing preference for natural tyrosinase inhibitors, especially those from botanical drugs and their metabolites, due to their perceived safety and consumer acceptance. A number of the tyrosinase inhibitors in use today have been isolated from various parts of botanical drugs, such as seeds, roots, and leaves. Botanical drug extracts, rich in bioactive metabolites, have long been employed for cosmetic and therapeutic purposes. Essential oils, as important functional metabolites, are usually valued for their antimicrobial and antioxidant properties, often making them as natural preservatives in cosmetic and medicine industries (Hazafa et al., 2020; Carqueijeiro et al., 2020; Li et al., 2020).

*Rosa × damascena* Herrm., commonly known as the Damask rose, is a significant species within the *Rosa genus*, which encompasses at least 200 species (Mahboubi, 2016). Many researches have demonstrated the presence of numerous metabolites in this plant with potential pharmacology properties (Lee et al., 2016). Now, *Rosa × damascena* Herrm. essential oil (RDEO) has been recognized for its high economic value, notably in cosmetic industry (Ho et al., 2020; Cebi, 2021). *Rosa × damascena* Herrm. and its essential oil RDEO are well-documented their antidiabetic, antioxidant, anti-aging, and anti-inflammatory properties (Akram et al., 2020; Al-Oqail et al., 2021). Previous reports found that citronellol and geraniol were its main active metabolites (Xiao et al., 2023).

As far as we know, the anti-tyrosinase activity and potential mechanism of RDEO had not been detailed investigated. Thence, in this study, we delved into the anti-tyrosinase properties of RDEO and analyzed phytochemical compositions.

## 2 Results and discussion

### 2.1 Inhibitory activity

**FIGURE 1:**
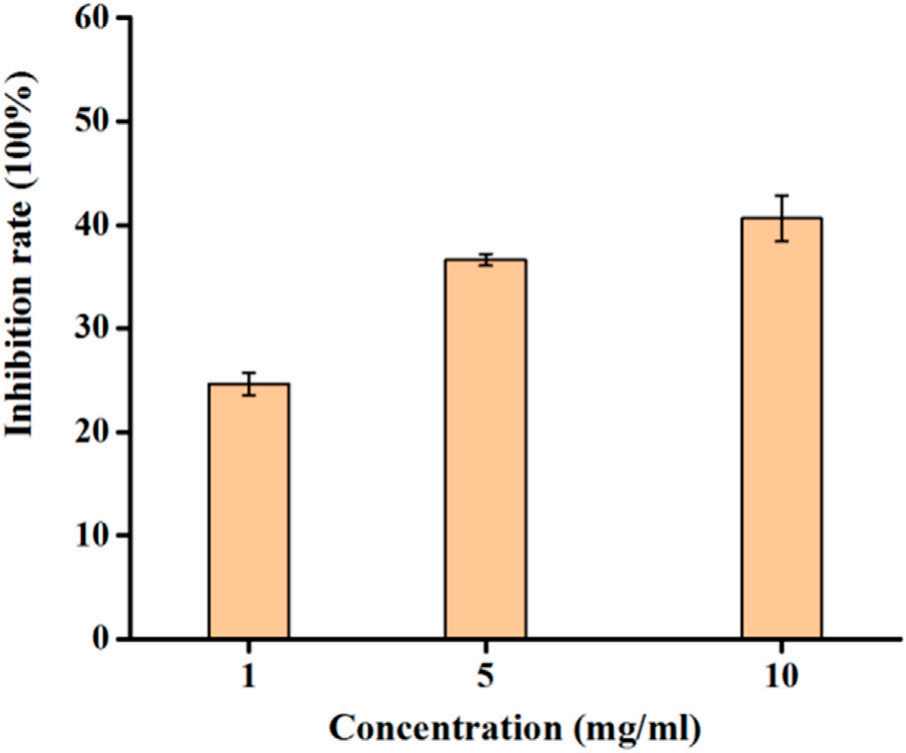
Inhibition rate of RDEO on tyrosinase.

The study delved into the inhibitory effects of RDEO on tyrosinase, an enzyme pivotal in melanin biosynthesis. In our experiments (Figure 1), we observed that RDEO exhibited a concentration-dependent inhibitory effect on tyrosinase activity. RDEO, with concentrations ranging from 1 to 10 mg/mL, obviously reduced the tyrosinase activity. Although, RDEO displayed lower tyrosinase inhibitory than kojic acid (IC_50_ = 2.2 ± 0.3 mg/L), RDEO still was proved to be one natural tyrosinase inhibitor. This finding is particularly promising given the increasing demand for natural metabolites to synthetic skin lightening agents.

### 2.2 Inhibition kinetics

**FIGURE 2:**
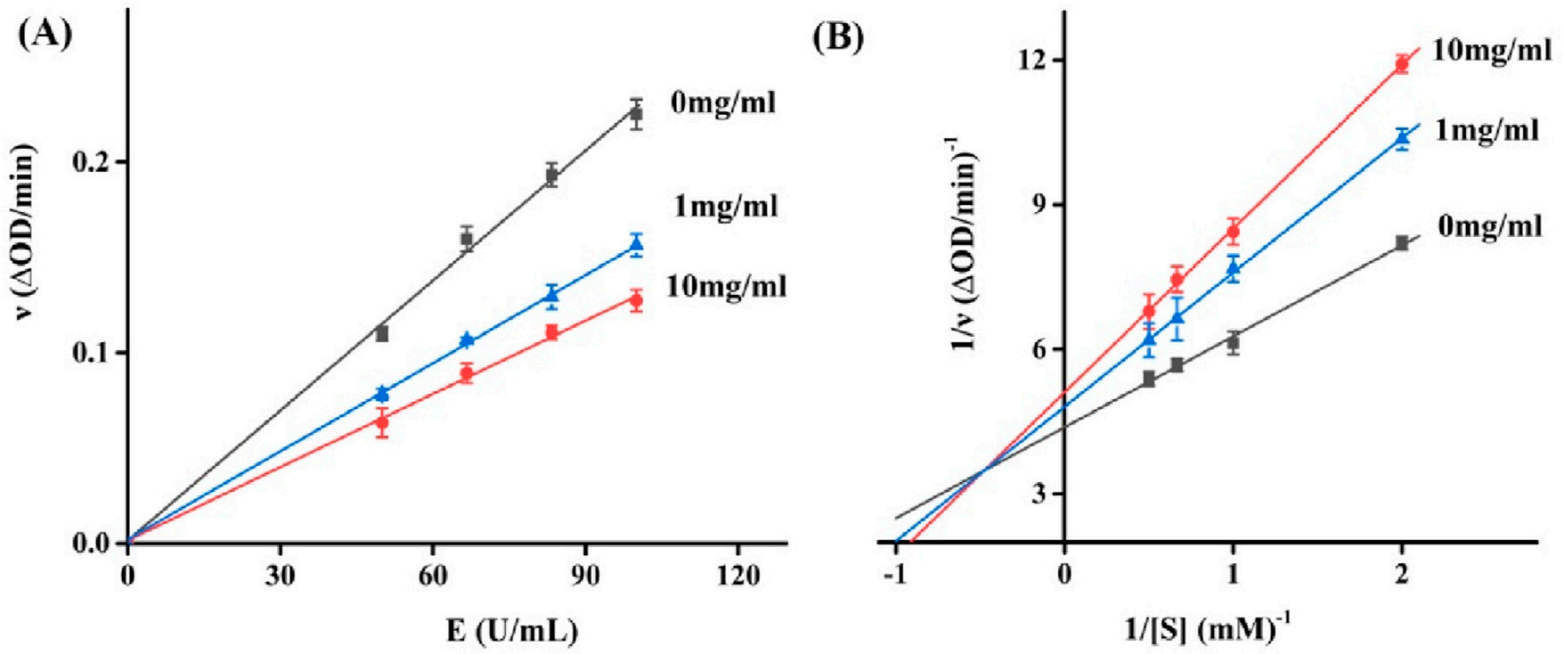
**(A,B)** Inhibition kinetics assay of RDEO on tyrosinase.

Aiming to elucidate the mechanism by which RDEO impedes tyrosinase activity. The kinetic study between RDEO and tyrosinase offers intriguing insights into the nature of their molecular relationship. Enzyme inhibitors are generally divided into reversible inhibitors and irreversible inhibitor according to the binding degrees of inhibitors with enzymes, which can be judged by the effects of inhibitors on enzymatic reactions. Reversible inhibitor can bind to enzymes or enzyme substrate complexes through non-covalent bonds, while irreversible inhibitors can bind to certain essential functional groups in enzyme active center through covalent bonds. In Figure 2A, catalytic rates (△OD/min) of tyrosinase were measured under RDEO (0–10 mg/mL), which revealed that RDEO lines (0–10 mg/mL) passed origin point with reducing slopes. The results indicated that RDEO cannot completely inactivate the efficacy enzyme, but only reduce its catalytic rate, revealing RDEO as a typical reversible inhibitor (Xu et al., 2020; Li et al., 2023; Xiao et al., 2023). In addition, reversible inhibitors are divided into competitive inhibitors, non-competitive inhibitors, and mixed-type inhibitors. In Figure 2B, the Lineweaver-Burk plots of catalytic rates (△OD/min) were obtained under RDEO (0–10 mg/mL). The lines intersected in the second quadrant and the slops increased with RDEO concentrations, which indicated that RDEO inhibited tyrosinase in a mixed-type (Zhang et al., 2022; Wu et al., 2023; Li et al., 2024a). This suggested that RDEO could bind to both the free enzyme and the enzyme-substrate complex, thereby influencing the enzyme’s activity.

### 2.3 Inhibition constant

**FIGURE 3:**
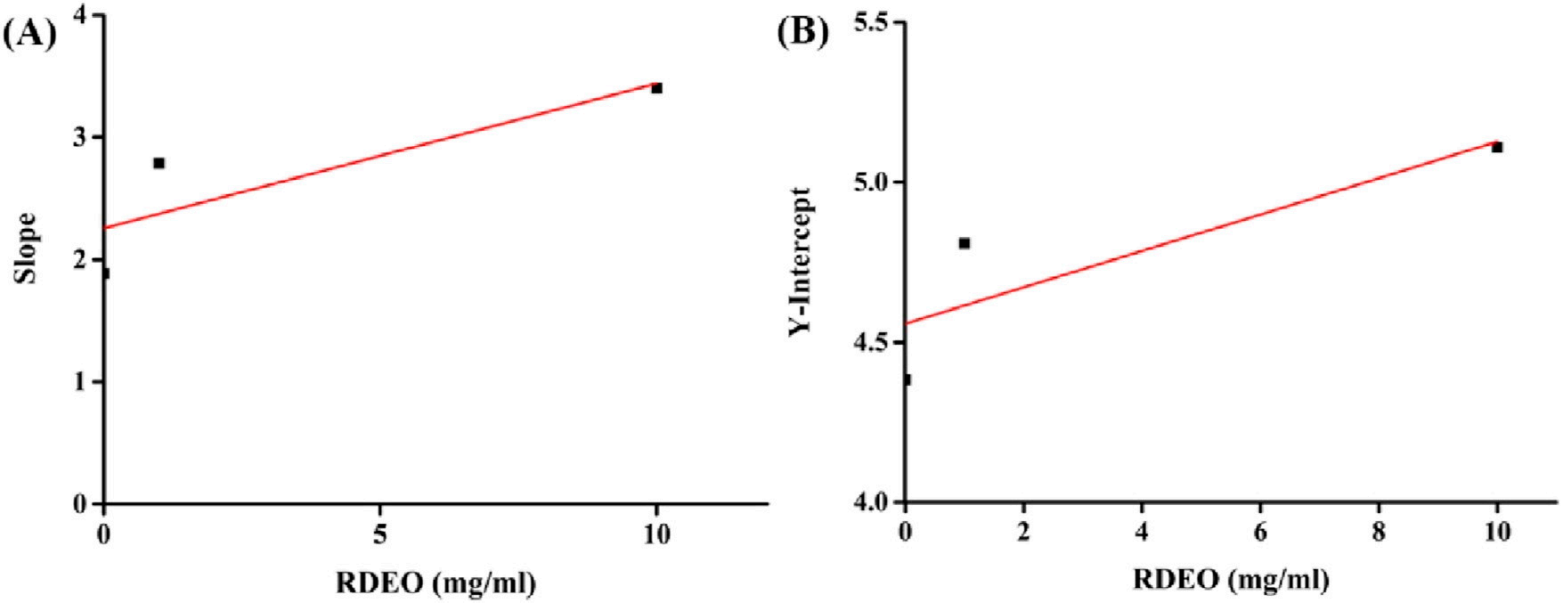
**(A,B)** K_i_ and K_is_ of RDEO on tyrosinase.

The analysis of the secondary curves (Figures 3A, B) derived from our kinetic data yielded inhibition constants, Ki (19.1 mg/mL) and Kis (80.1 mg/mL) from the plots of lines slop and intercept against RDEO concentration, respectively. The lower K_i_ value in comparison to K_is_ indicated a stronger binding affinity of RDEO to free enzyme (Deng et al., 2022; Feng et al., 2024; Min et al., 2024).

### 2.4 CD assay

**FIGURE 4:**
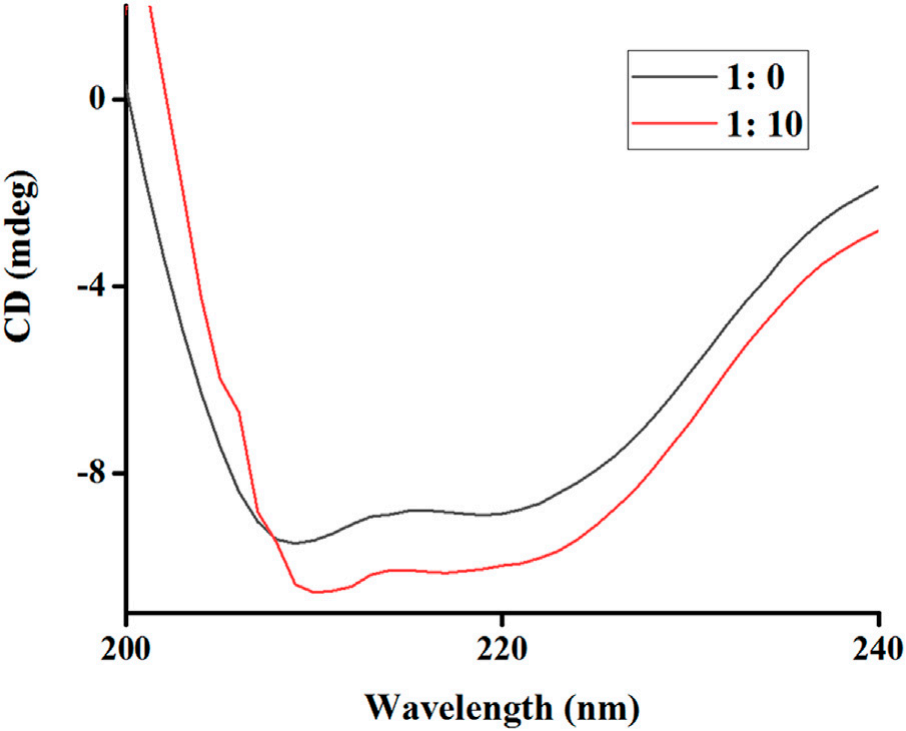
CD spectra of tyrosinase-RDEO.

This section aimed to assess the conformational changes in tyrosinase that might result from its interaction with RDEO, which could elucidate the underlying mechanism of its inhibitory effect. Our results from the CD assay (Figure 4) demonstrated that RDEO (ratio: 10: 1) induced alterations in characteristic band (205–226 nm) of tyrosinase, which was indicative of its α-helical content. The treatment with RDEO led to noticeable changes in both intensity and shape of bands, suggesting a direct influence on the enzyme’s secondary structure.

Further analysis of the CD spectra (Table 1) revealed that RDEO (ratio: 10: 1) treatment resulted in a decrease in α-helix, β-turn, and random coil contents, and the increase in β-sheet content. These structural changes were believed to contribute to the reduction in tyrosinase’s activity, providing a plausible explanation for the observed inhibition. The CD assay findings underscored the potential of RDEO as a modulator of tyrosinase conformation and activity, supporting the use of RDEO in the development of novel skin lightening agents.

**TABLE 1:** Structural contents of tyrosinase by RDEO.

Ratio	α-Helix (%)	β-Sheet (%)	β-Turn (%)	Random coil (%)
1: 0	8.8	55.5	19.6	36.0
1: 10	8.6	55.7	18.6	35.8

### 2.5 Intracellular assay

**FIGURE 5:**
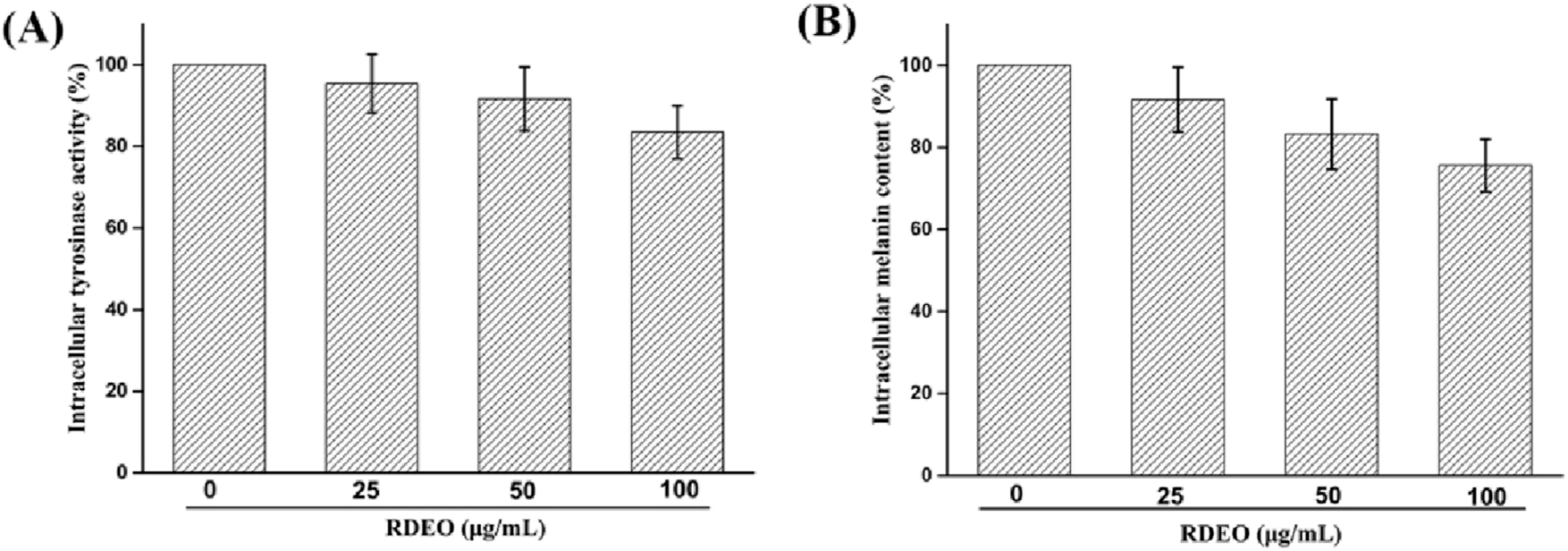
**(A,B)** Effects of RDEO on intracellular tyrosinase activity and melanin content.

Finally, we determined the effects of RDEO on intracellular tyrosinase activity and melanin content in B16F10 cells. As shown in Figure 5A, RDEO (25–100 μg/mL) treatment would result in the reduction of intracellular tyrosinase activity in dose-manner, suggesting its inhibitory efficacy on intracellular tyrosinase. This result was consistent with that of *in vitro* tyrosinase inhibitory of RDEO. Parallel to tyrosinase activity assessment, cells melanin content was quantified and found that RDEO (25–100 μg/mL) treatment also caused the reduction of intracellular melanin content (Figure 5B). Thence, RDEO showed a certain ability to resist melanin production, mainly due to its inherent ability to inhibit tyrosinase activity.

### 2.6 Phytochemical composition analysis

**FIGURE 6:**
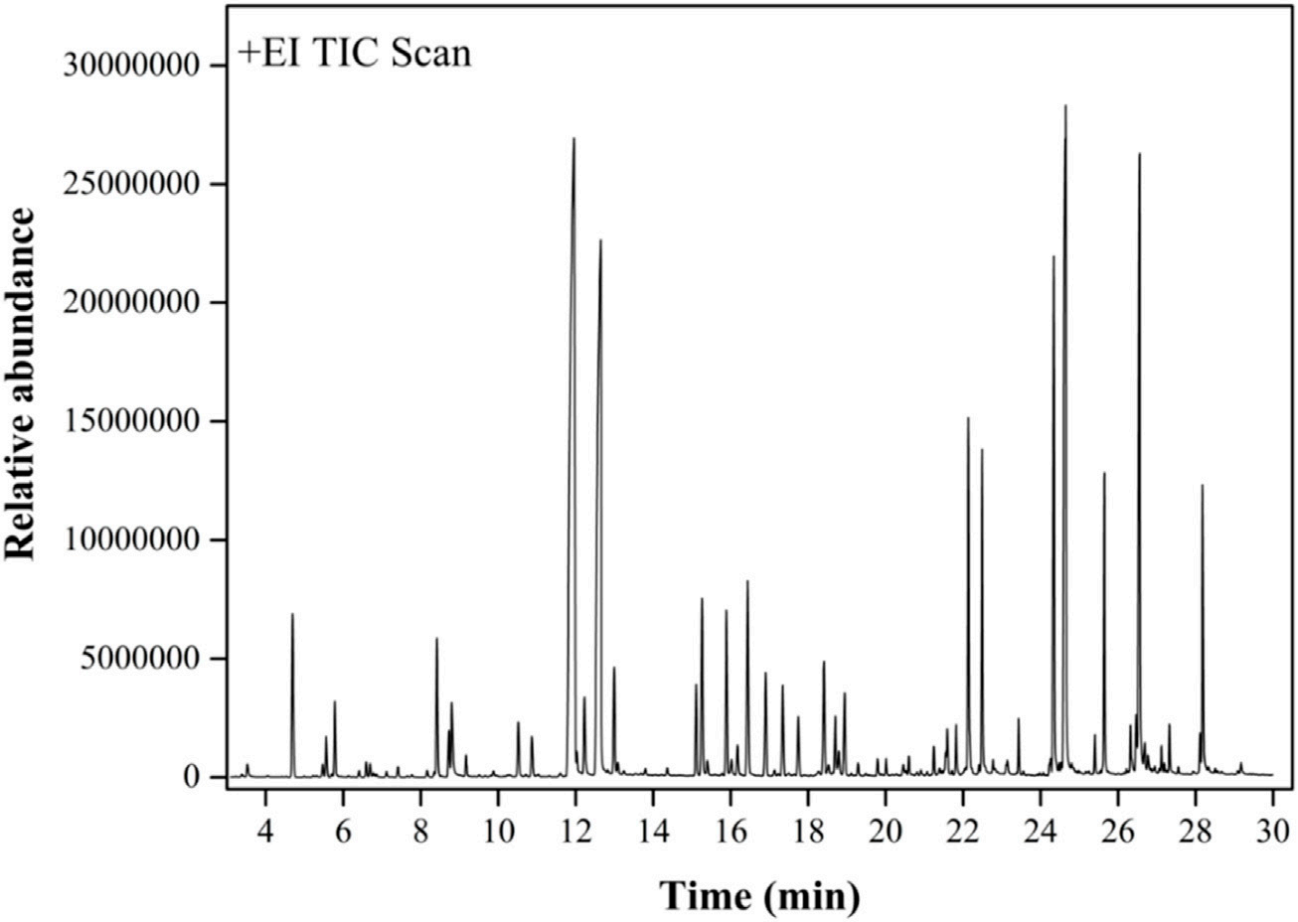
GC-MS results of RDEO.

Above investigation not only provided a deeper understanding of the inhibitory effects of RDEO on tyrosinase but also set the stage for future research into the specific compounds responsible for this activity. This section aimed to dissect the complex mixture of volatile metabolites presenting in RDEO and identify the metabolites that might contribute to its biological effects. Utilizing gas chromatography-mass spectrometry (GC-MS), we meticulously characterized the chemical composition of RDEO (Figure 6). A total of 66 metabolites were identified from RDEO (Table 2).

**TABLE 2:** Analyzed metabolites in RDEO.

No.	Metabolite	Peak RT (min)	Content (%)
1	1-Hexanol	3.527	0.14
2	L-α-Pinene	4.692	1.73
3	(+)-Sabinene	5.465	0.13
4	L-β-Pinene	5.56	0.41
5	Myrcene	5.782	0.76
6	o-Cymene	6.587	0.15
7	Cyclohexene, 1-methyl-5-(1-methylethenyl)-(R)	6.688	0.15
8	Linalool	8.42	1.48
9	cis-Rose oxide	8.727	0.46
10	Phenethyl alcohol	8.801	1.02
11	(2R,4R)-rose oxide	9.172	0.22
12	Terpinen-4-ol	10.517	0.62
13	3-Cyclohexene-1-methanol, α,α,4-trimethyl-(R)	10.871	0.48
14	Citronellol	11.952	21.22
15	(3Z)-3,7-Dimethyl-3,6-octadien-1-ol	12.031	0.3
16	Citral	12.227	0.93
17	Geraniol	12.645	14.1
18	α-Citral	12.993	1.11
19	Citronellyl Formate	13.085	0.13
20	Citronellyl acetate	15.113	0.98
21	2-methoxy-5-prop-2-enyl-phenol	15.261	2.08
22	Nerol acetate	15.399	0.17
23	Geranyl acetate	15.891	1.79
24	β-bourbonene	16.018	0.23
25	β-Elemen	16.177	0.35
26	Methyl eugenol	16.442	2.24
27	β-Caryophyllene	16.903	1.21
28	α-guaiene,guaia-1 (5),11-diene, [1S-(1a,4a,7a)]-1,2,3,4,5,6,7,8-octahydro-1,4-dimethyl-7-(1-methylethenyl)-azulene	17.348	1.01
29	1,4,7-Cycloundecatriene, 1,5,9,9-tetramethyl-	17.745	0.67
30	Tricyclo [4.4.0.02,7]decane,1-methyl-3-methylene-8-(1-methylethyl)-, (1R,2S,6S,7S,8S)-rel	18.412	1.27
31	β-selinene	18.528	0.14
32	Pentadecane	18.698	0.6
33	cis-β-Guaiene	18.793	0.28
34	α-Bulnesene	18.942	0.9
35	Naphthalene,1,2,3,5,6,8a-hexahydro-4,7-dimethyl-1-(1-methylethyl)	19.291	0.13
36	Elemol	19.794	0.16
37	Nerolidol	20.011	0.15
38	Caryophyllene oxide	20.461	0.13
39	Hexadecane	20.599	0.17
40	2-Naphthalenemethanol, 1,2,3,4,4a,5,6,7-octahydro-α,α,4a,8-tetramethyl-, (2S,4aR)	21.245	0.27
41	β-Eudesmol	21.552	0.24
42	(1aR,7aα,7bβ)-Decahydro-1,1,3aβ,7-tetramethyl-1H-cyclopropa [a]naphthalen-7α-ol	21.594	0.47
43	3-Heptadecene, (Z)	21.817	0.53
44	Heptadecane	22.14	3.3
45	2,6,10-Dodecatrien-1-ol, 3,7,11-trimethyl	22.495	2.88
46	2,6,10-Dodecatrienal, 3,7,11-trimethyl	22.775	0.13
47	(E)-7-Octadecene	23.146	0.14
48	Octadecane	23.437	0.4
49	(9Z,12Z)-Phenethyl octadeca-9,12-dienoate	24.258	0.23
50	1-Nonadecene	24.343	5.16
51	Z-5-Nonadecene	24.48	0.12
52	1-Heneicosanol	24.533	0.13
53	Eicosane	24.65	11.03
54	12-Methyl-E,E-2,13-octadecadien-1-ol	24.809	0.17
55	1-Eicosanol	25.397	0.31
56	Eicosane	25.645	2.35
57	heneicos-1-ene	26.323	0.54
58	2,2-Dimethoxypropane	26.456	0.61
59	Heneicosane	26.556	6.65
60	2-Octen-1-ol, 3,7-dimethyl	26.689	0.3
61	4,8-Decadienal, 5,9-dimethyl	26.773	0.18
62	6,11-Dimethyl-2,6,10-dodecatrien-1-ol	27.118	0.21
63	Docosane	27.324	0.34
64	9-Tricosanol, acetate	28.113	0.32
65	Tricosane	28.182	2.64
66	Pentacosane	29.172	0.16

**FIGURE 7:**
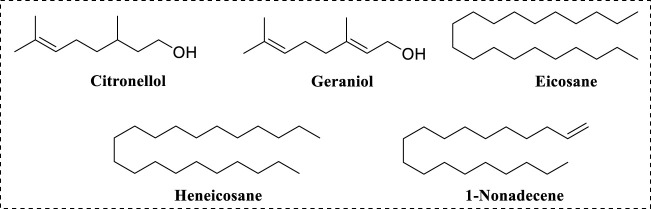
Chemical structures of main metabolites in RDEO.

The major compositions of RDEO were citronellol (21.22%) and geraniol (14.1%), eicosane (11.03%), heneicosane (6.65%) and 1-nonadecene (5.16%), respectively (Figure 7). In one previous work about RDEO (Xiao et al., 2023), citronellol, geraniol, eicosane, heneicosane, 1-nonadecene were also confirmed as the main components of RDEO, except the differences in their contents. In particular, geraniol was reported to have tyrosinase inhibitory activity (Lante and Tinello, 2015; Mahant et al., 2021). Phytochemical composition analysis of RDEO had shed light on its complex chemical landscape. The identification of these metabolites set the stage for further research into their individual and combined effects on tyrosinase.

## 3 Conclusion

*Rosa × damascena* Herrm. essential oil (RDEO), as important functional metabolites, was widely known due to its biological activities. Therefore, in this paper, the tyrosinase inhibitory effects of RDEO and its phytochemical compositions were analyzed. Activity screening results showed that RDEO exhibited anti-tyrosinase activity and was a reversible and mixed inhibitor. CD assay results revealed that RDEO could affect the conformation of tyrosinase. GC-MS analysis of RDEO found that citronellol (21.22%), geraniol (14.1%), eicosane (11.03%), heneicosane (6.65%) and 1-nonadecene (5.16%) were the main compositions. In particular, geraniol was reported to have tyrosinase inhibitory activity. In B16F10 cells, RDEO (25–100 μg/mL) could inhibit intracellular tyrosinase activity and decrease melanin content. This study provided data support for RDEO as a natural tyrosinase inhibitor and its applications in cosmetics and medicine.

## 4 Materials and methods

### 4.1 Rosa × damascena Herrm. essential oil

Rosa × damascena Herrm. essential oil (RDEO) was obtained from Natural Laboratory Bul Rose Ltd. (batch number: T′02, date of production: June 2023) (Heinrich et al., 2020; Heinrich et al., 2022). Brief preparation was as follows using steam distillation method. 100 g of dry damask rose powder and 2.5 L distilled water were added into a distillation flask (5 L) and heated to boil. The obtained oil-water mixture was layered and dried using anhydrous sodium sulfate to produce RDEO.

### 4.2 Tyrosinase activity

RDEO was assayed its anti-tyrosinase activity using mushroom tyrosinase (Li et al., 2023). RDEO was dissolved in DMSO for subsequent test. RDEO (10 μL) was added into tyrosinase (130 μL) and L-DOPA (50 μL). Then, the mixture was measured the absorbance at 475 nm. Inhibition rate was calculated compared to black.

### 4.3 Inhibitory kinetic

Inhibitory kinetics of RDEO on tyrosinase was examined to delineate the mode of inhibition. The assay involved varying concentrations of tyrosinase and substrate in the presence of RDEO. The changes in absorbance were monitored, and Lineweaver-Burk plots were constructed to analyze the data (Lu et al., 2023a; Lin et al., 2023).

### 4.4 CD spectra

CD spectra were employed in this study to investigate the conformational changes in tyrosinase upon interaction with RDEO (Lu et al., 2023b). CD spectra were recorded, following the addition of RDEO to the tyrosinase. The spectra were captured in the wavelength range of 190–260 nm. The ratios of tyrosinase to RDEO were varied to 1: 0 and 1: 10.

### 4.5 Intracellular assay

Intracellular tyrosinase activity: B16F10 cells were treated with RDEO (25–100 μg/mL) for 24 h and then lysed using lysis buffer. Subsequently, an appropriate amount of L-DOPA was added and measured its absorbance at 457 nm.

Intracellular melanin content: Intracellular tyrosinase activity: B16F10 cells were treated with RDEO (25–100 μg/mL) for 24 h, treated in NaOH solution at 100°C for 1 h, and measured its absorbance at 405 nm.

### 4.6 Phytochemical composition

Phytochemical composition analysis of RDEO (dissolution in methanol) was conducted on GC-MS. GC conduction (Hp-5MS quartz capillary column (inner diameter 30 m × 0.25 mm, film thickness 0.25 μm)) was as follow: Initial temperature was 70°C for 2 min, followed by rate of 5°C/min to 150°C for 16 min, rate of 10°C/min to 220°C for 7 min, and rate of 10°C/min to 220°C for 2 min. MS detection: ionization energy: 70 eV, ion source temperature: 230°C; scanning mass range: 40–500 m/z, helium (He) flow rate: 1.0 mL/min. Compounds were identified using standard spectral library.

### 4.7 Statistical analysis

Data were presented as mean ± SD and evaluate the differences suing One-way ANOVA. *P* < 0.05 was considered significant (Hu et al., 2024; Li et al., 2024b; Liang et al., 2024).

## Data Availability

The original contributions presented in the study are included in the article/supplementary material, further inquiries can be directed to the corresponding authors.
